# Oral microbial dysbiosis in patients with periodontitis and chronic obstructive pulmonary disease

**DOI:** 10.3389/fcimb.2023.1121399

**Published:** 2023-02-09

**Authors:** Siqin Liu, Guofang Xie, Meifeng Chen, Yukun He, Wenyi Yu, Xiaobo Chen, Weigang Mao, Nanxia Liu, Yuanjie Zhang, Qin Chang, Yingying Qiao, Xinqian Ma, Jianbo Xue, Mengtong Jin, Shuming Guo, Yudong Hou, Zhancheng Gao

**Affiliations:** ^1^ School of Stomatology, Binzhou Medical University, Yantai, China; ^2^ Department of Stomatology, Linfen Central Hospital, Linfen, China; ^3^ Department of Respiratory and Critical Care Medicine, Linfen Central Hospital, Linfen, China; ^4^ Department of Respiratory and Critical Care Medicine, Peking University People’s hospital, Beijing, China; ^5^ Department of Science and Education, Linfen Central Hospital, Linfen, China; ^6^ Nursing department, Linfen Central Hospital, Linfen, China

**Keywords:** periodontal disease, COPD, oral microbiome, 16S rRNA, subgingival plaque, gingival crevicular fluid, inflammation, chronic obstructive pulmonary disease

## Abstract

**Background:**

Oral microbiota is closely related to the homeostasis of the oral cavity and lungs. To provide potential information for the prediction, screening, and treatment strategies of individuals, this study compared and investigated the bacterial signatures in periodontitis and chronic obstructive pulmonary disease (COPD).

**Materials and methods:**

We collected subgingival plaque and gingival crevicular fluid samples from 112 individuals (31 healthy controls, 24 patients with periodontitis, 28 patients with COPD, and 29 patients with both periodontitis and COPD). The oral microbiota was analyzed using 16S rRNA gene sequencing and diversity and functional prediction analysis were performed.

**Results:**

We observed higher bacterial richness in individuals with periodontitis in both types of oral samples. Using LEfSe and DESeq2 analyses, we found differentially abundant genera that may be potential biomarkers for each group. *Mogibacterium* is the predominant genus in COPD. Ten genera, including *Desulfovibrio*, *Filifactor*, *Fretibacterium, Moraxella, Odoribacter, Pseudoramibacter Pyramidobacter, Scardovia, Shuttleworthia* and *Treponema* were predominant in periodontitis. *Bergeyella, Lautropia, Rothia, Propionibacterium* and *Cardiobacterium* were the signature of the healthy controls. The significantly different pathways in the Kyoto Encyclopedia of Genes and Genomes (KEGG) between healthy controls and other groups were concentrated in genetic information processing, translation, replication and repair, and metabolism of cofactors and vitamins.

**Conclusions:**

We found the significant differences in the bacterial community and functional characterization of oral microbiota in periodontitis, COPD and comorbid diseases. Compared to gingival crevicular fluid, subgingival plaque may be more appropriate for reflecting the difference of subgingival microbiota in periodontitis patients with COPD. These results may provide potentials for predicting, screening, and treatment strategies for individuals with periodontitis and COPD.

## 1 Introduction

The oral microenvironment is complicated and comprises more than 700 bacterial species (Dewhirst et al., 2010). Among them, 400 species have been identified in periodontal pockets. Oral microbial dysbiosis is known to impact chronic inflammatory diseases (Thomas et al., 2021). Microbial migration from the oral cavity appears to be a significant source of the lung microbiome through microaspiration and inhalation (Bassis et al., 2015). Thus, the oral microbiota is closely related to the homeostasis of the oral cavity and lungs.

Periodontitis , a chronic infectious disease caused by periodontal pathogens, is characterized by the loss of gingiva, bone, and ligament and deep periodontal pockets between the tooth and gingiva (Kinane et al., 2017). Periodontitis is a highly prevalent oral disease in China, with a prevalence of up to 52.8% (Jiao et al., 2021). Emerging evidence has revealed that periodontitis is closely related to the oral microbiota, which can increase the risk of the development of chronic inflammatory conditions, thereby leading to coronary artery disease, systemic lupus erythematosus, and respiratory disease (Gomes-Filho et al., 2010; Preshaw et al., 2012; Slocum et al., 2016; Li et al., 2020). Chronic obstructive pulmonary disease (COPD) is one of the most common respiratory diseases characterized by progressive and non-reversible airflow limitation (Barnes et al., 2015a). Recurrent episodes of exacerbations in COPD lead to significant mortality worldwide (Barnes et al., 2015b; Caramori et al., 2016; Rabe and Watz, 2017). Disturbed lung microbiome and abnormal inflammatory reactions are the two main causes of acute exacerbation of COPD (Mammen and Sethi, 2016).

Gram-negative bacteria, such as *Porphyromonas gingivalis*, *Treponema denticola*, and species are believed to be the main oral microbiome in the periodontal inflammatory response (Gaeckle et al., 2020). Compared with the control group, the abundance of *P.gingivalis*, *Klebsiella pneumoniae*, *Pseudomonas aeruginosa* and *Streptococcus pneumoniae* increased in participants with COPD (Tan et al., 2019). Moreover, *Veillonella*, *Rothia*, and *Actinomyces* were more enriched in patients with COPD and periodontitis than in HCs(Lin et al., 2020). Treating periodontitis significantly reduced exacerbation frequency in patients with COPD (Kucukcoskun et al., 2013). Although most recent studies have explored the relationship and influence mechanism of periodontitis or COPD, research on the alteration of the oral microbiome in patients with periodontitis, COPD or both, remains insufficient. Moreover, previous studies have mainly focused on saliva samples; however, the bacterial composition differs between saliva and subgingival pockets (Jakubovics and Kolenbrander, 2010; Jia et al., 2018). As the main accumulation site of periodontal pathogens, subgingival plaque more directly reflects the status of the subgingivalmicrobiome.

In this study, we investigated the shared and specific alterations in the oral microbiomes of participants with periodontitis, COPD, or both, through 16S rRNA gene sequencing.

## 2 Material and methods

### 2.1 Study participants

The present study was approved by the ethics committee of Linfen Central Hospital (Ethics Approval No. 2021-42-1) and was performed in accordance with the Declaration of Helsinki. Written informed consent was obtained from all participants prior to clinical data collection and sampling.

A total of 112 participants were recruited at Linfen Central Hospital, including 31 healthy controls (HC group), 24 periodontitis patients without COPD (P group), 28 COPD patients without periodontitis(COPD group), and 29 patients with both periodontitis and COPD(P_COPD group). The diagnosis and assessment of the severity of COPD were made according to the recommendations of the Global Initiative for Chronic Obstructive Lung Disease (GOLD) committee (Vogelmeier et al., 2017). The diagnosis and assessment of the periodontitis were based on the new classification, Classification of Periodontal and Peri-implant Diseases and Conditions (Tonetti et al., 2018). Other inclusion criteria included: (1) aged ≥18 years; and (2) Periodontitis from stage II to IV, grade B. The exclusion criteria were antibiotic using before during the last three months, other systemic diseases, administration of periodontal therapy during the last three months (Cai et al., 2021). General participant demographics, including age, gender, blood routine records, pulmonary function test results and clinical treatments were collected from medical record system using a standard form.

### 2.2 Sample collection

Before sample collection, the participants were asked to rinse their mouth for removing the food residues and debris. We obtained oral samples from four first incisor teeth and four first molar teethof each participant. The first molars and incisors are the main sites of periodontal lesions, and we selected 11, 21, 31, 41, 16, 26, 36, 46 as the main sampling sites based on previous periodontal microbiology studies (Zhou et al., 2020). Clinical examination was performed before sampling to ensure that the sampling site clinical attachment loss (CAL) ≥ 3mm, probing depth (PD) ≥ 4mm and bleeding on probing. If one of these teeth was missing, the adjacent tooth was collected. After drying the target sites, gingival crevicular fluid (GCF) samples were collected with sterile absorbent paper points from gingival sulcus of each tooth. After removal of supragingival plaque, subgingival plaque (SP) samples were collected with sterile Gracey curettes from the buccal and lingual sides of each tooth. The sample of each participant was collected in the eppendorf tube. All oral specimens (subgingival plaque and gingival crevicular fluid) were stored in -80°C until DNA extraction.

### 2.3 DNA extraction, 16S rRNA gene amplification, and sequencing

Total bacterial DNA was extracted from oral samples using SteadyPure Bacterial Genomic DNA Extraction Kit(Accurate Biotechnology(Hunan)Co,Ltd,China) following the manufacturer’s instructions. Hypervariable regions (V2, V3, V4, V6-7, V8 and V9) of the 16S rRNA were amplified using two primer sets in the Ion 16STM Metagenomics Kit (ThermoFisher Scientific, UK). XP beads were used to purify the amplification products and quantified by Qubit4 (ThermoFisher Scientific, USA). Purified amplicons were ligated with barcodes and then generated for the libraries. Then the libraries were pooled in equimolar amounts on chip 530 and sequenced to single-end, 250-base-pair reads on an Ion GeneStudio S5 System (ThermoFisher Scientific, USA) based on Ion Reporter metagenomics workflow (Ion 16S mNGS). Quality filtering, trimming and dereplication of raw sequencing reads were conducted automatically on Ion Reporter metagenomics workflow, relying on default parameters. Unaligned binary data files (Binary Alignment Map, BAM) generated by the Ion Torrent PGM were uploaded to Ion Reporter and analyzed using default settings (Malczynski et al., 2021).

### 2.4 Statistical analysis

Quantitative variables conforming to normal distribution were presented as the mean ± SD analyzed by Student’s t test and analysis of variance (ANOVA), while Quantitative variables of non-normal distribution were presented as median and interquartile ranges (25th and 75th percentiles) and analyzed by Mann-Whitney U or Kruskal-Wallis test. Categorical variables were presented as rate or percentage, and chi-square test or Fisher test were used to analysis. The alpha diversity was evaluated using the Chao-1, Shannon, abundance-based coverage estimator (ACE) and Simpson indices, respectively. The beta diversity has been evaluated through principal coordinates analysis (PCoA) ordination of variance and compared using Bray-Curtis dissimilarity. Differential species among groups was explored with the linear discriminant analysis (LDA) effect size (LEfSe) method (Shi et al., 2021) and DESeq2 analysis (Lu et al., 2022). The microbiome phenotypes were predicted by BugBase (Ward et al., 2017). The BugBase phenotype predictions were implemented using the online web page https://bugbase.cs.umn.edu/index.html. Prediction of the abundances of functional categories was conducted using PICRUSt2 (Douglas et al., 2020). Statistics and visualization of functional data were depicted using STAMP (Chowdhry et al., 2018). *P*<0.05 was considered as statistically significant.

## 3 Results

### 3.1 Clinical characteristics of the study population

A total of 112 participants were enrolled in our study, and the basic characteristics of each group are listed in **Table 1**. There were no differences among the groups except for gender, age and smoking. The healthy control (HC) group’s median age was significantly younger than the diseased groups. The median age of the comorbid (P_COPD) group was highest. Moreover, the healthy group had a higher proportion of female participants. Significant difference of smoking was only existed between healthy control group and periodontitis group. Gender, age and smoking status were treated as confounding factors which were corrected in the difference analysis(**Figure S4A**). There were no significant differences in the GOLD grade and clinical indicators between the COPD and P_COPD groups.

**Table 1:** Demographical and Clinical Features of Included Subjects.

	HC(n=31)	P(n=24)	COPD(n=28)	P_COPD(n=29)	*p*-value
Age	25(23-38)	53.5(47.25-61.25)	61(51.75-67.75)	66(60.5-72.5)	<0.005^a^
Gender,n (%)					<0.001^b^
female	19(61.3%)	9(37.5%)	5(17.9%)	4(13.8%)	
male	12(38.7%)	15(62.5%)	23(82.1%)	25(86.2%)	
Somkers,n (%)					
Current smoker	3(9.7%)	12(50%)	7(25%)	7(24.1%)	<0.001^c^
Former smoker	0	0	7(25%)	15(51.7%)	>0.05
Nonsmoker	28(90.3%)	12(50%)	14(50%)	7(24.1%)	<0.001^d^
PD(mm)	2(1-2)	6.33(4.33-6.92)	2(1.25-2)	6.33(5-6.67)	<0.001^e^
BOP%	2.08(2.08-4.17)	78.13(71.35-83.33)	2.08(2.08-4.17)	81.25(68.75-87.5)	<0.001^f^
Stage(%)					0.612
II	—	9(37.5%)	—	8(27.6%)	
III	—	10(41.7%)	—	16(55.2%)	
IV	—	5(20.8%)	—	5(17.2%)	
GOLD(%)					0.712
I	—	—	3(10.7%)	6(20.7%)	
II	—	—	11(39.3%)	10(34.5%)	
III	—	—	9(32.1%)	7(24.1%)	
IV	—	—	5(17.9%)	6(20.7%)	
BMI(kg/m^2^)	—	—	27.35(23.43-29.3)	23.5(22.1-25.75)	0.007
FVC(L)	—	—	2.88(2.51-3.56)	2.82(2.34-3.67)	0.943
FEV1(L)	—	—	1.43(0.99-2.18)	1.34(0.92-2.12)	0.472
FEV1%	—	—	58.31(35.71-73.54)	53.08(33.56-75.52)	0.576
Peripheral blood					
WBC(x10^9^/L)	—	—	6.3 ± 2.1	6.5 ± 1.7	0.675
RBC(x10^12^/L)	—	—	4.7 ± 0.4	4.7 ± 0.4	0.797
HGB(g/L)	—	—	143.4 ± 11.2	142.8 ± 12.1	0.848
Neutrophil percentages(%)	—	—	57.2 ± 8.1	58.6 ± 10.4	0.578
Lymphocyte percentages(%)	—	—	31.1 ± 6.7	30.5 ± 9.9	0.799
Monocytes percentages(%)	—	—	7.5(6.45-10)	7.7(6.65-9)	0.958
Eosinophil percentages(%)	—	—	2.3(1.45-3.85)	2.3(1.25-2.85)	0.409
Basophil percentages(%)	—	—	0.5 ± 0.3	0.5 ± 0.2	0.706
Neutrophil(x10^9^/L)	—	—	2.99(2.62-5.125)	3.8(2.675-4.67)	0.482
Lymphocyte(x10^9^/L)	—	—	1.9 ± 0.5	2.0 ± 0.7	0.719
Monocytes(x10^9^/L)	—	—	0.43(0.375-0.565)	0.54(0.48-0.615)	0.147
Eosinophil(x10^9^/L)	—	—	0.15(0.08-0.22)	0.12(0.08-0.205)	0.567
Basophil (x10^9^/L)	—	—	0.03(0.02-0.04)	0.03(0.02-0.05)	0.333

^a^Significant difference exists among healthy control group and other groups, significant difference exists between periodontitis group and COPD with periodontitis group.

^b^Significant differences exists among healthy control group and other three groups.

^c,d^Significant difference exists between healthy control group and periodontitis group.

^e,f^Significant difference exists between healthy control group and periodontitis group, significant difference exists between COPD group and periodontitis group, significant difference exists between COPD group and COPD with periodontitis group.

BMI, body mass index; GOLD, grading of pulmonary function; WBC, white blood cell; RBC, red blood cell; PD, probing depth; BOP, bleeding on probing.

HC, health controls; P, patients with periodontitis; COPD, patients with chronic obstructive pulmonary disease; P_COPD, patients with comorbid diseases.

### 3.2 The oral microbial community in the periodontal pocket and crevice

**Figure 1:**
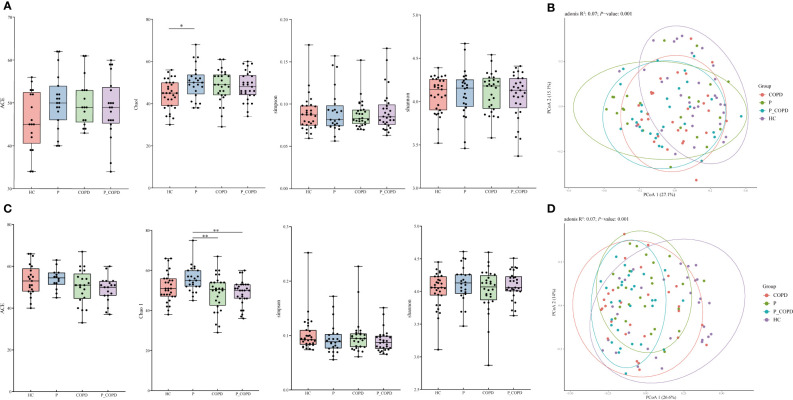
Alpha diversity analysis of healthy controls (HC), periodontitis (P) group, chronic obstructive pulmonary disease (COPD) group and comorbid diseases (P_COPD) group. Chao1, Shannon, abundance-based coverage estimator (ACE) and Simpson indices of each group, **(A)** in subgingival plaque samples and **(C)** in gingival crevicular fluid samples. Under Chao1 index, significant difference between HC and P was observed in subgingival plaque samples, significant differences between P and COPD, P and P_COPD were observed in gingival crevicular fluid samples. Principal coordinate analysis (PCoA) among healthy controls (HC), periodontitis (P) group, chronic obstructive pulmonary disease (COPD) group and comorbid diseases (P_COPD) group. **(B)** The PCoA plot showed a separation of samples from HC and other diseased groups in subgingival plaque samples. **(D)** The PCoA plot showed a separation of samples from HC and other diseased groups in gingival crevicular fluid samples. The samples of P_COPD were separated from P group.*: *p* < 0.05 **: *p* < 0.01.

All sequencing data for the four groups reached saturation at approximately 50,000 reads (**Figure S1**). For alpha diversity, the Chao1 index in the subgingival plaque (SP) samples from the periodontitis group was significantly higher than that in the HC group (**Figure 1A**, *P* = 0.0245). In the gingival crevicular fluid (GCF) samples, the Chao1 index of periodontitis group was significantly higher than that of the COPD group (**Figure 1C**, *P* = 0.0068) and P_COPD group (**Figure 1C**, *P* = 0.0063). However, no significant difference was found in the Shannon, Simpson and ACE indices among the four groups in the different sample types (**Figures 1A, C**, *P* > 0.05). To evaluate similarities among the four groups, PCoA was based on unweighted UniFrac distances. Regardless of the SP or GCF samples, beta diversity was different in the HC and diseased groups (**Figures 1B, D**). However, in the SP samples, the bacterial compositions in the periodontitis, COPD, and P_COPD groups were indistinguishable (**Figure 1B**, *P*>0.05). For the GCF samples, beta diversity was different between theperiodontitis and P_COPD groups (**Figure 1D**).

**Figure 2:**
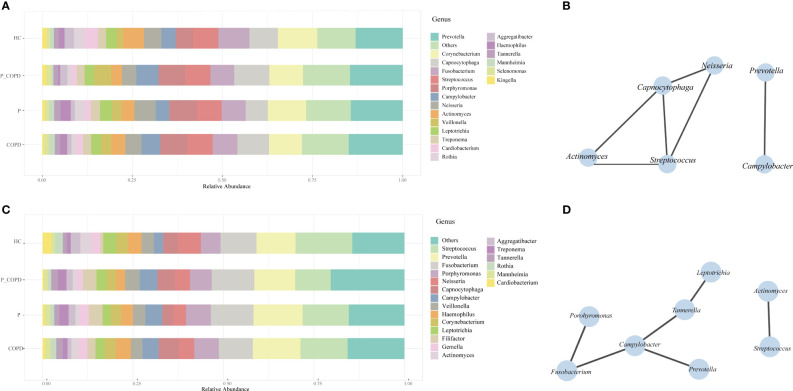
Relative abundances of the oral microbiota in healthy controls (HC), periodontitis (P) group, chronic obstructive pulmonary disease (COPD) group and comorbid diseases (P_COPD) group. Stacked bar plots showing relative abundances of the oral microbiota at the genus level **(A)** in subgingival plaque samples, **(C)** in gingival crevicular fluid samples. The correlation network analysis of the core microbiota based on SparCC. The core microbiota was defined as which covering 100% of all samples. **(B)** In subgingival plaque samples. **(D)** In gingival crevicular fluid samples.

Eight phyla, 50 families, 98 genera were detected in the SP samples. The most abundant genera were *Prevotella*, *Corynebacterium, Capnocytophaga*, *Fusobacterium*, *Streptococcus and Porphyromonas* (**Figure 2A**). *Actinomyces*, *Campylobacter, Capnocytophaga*, *Neisseria*, *Prevotella* and *Streptococcus* were present in all SP samples (**Figure 2B**). Nine phyla, 57 families and 118 genera were identified in the GCF samples. The most abundant genera were *Streptococcus*, *Prevotella*, *Fusobacterium*, *Porphyromonas*, *Neisseria* and *Capnocytophaga* (**Figure 2C**). The core microbiota of the GCF samples were *Actinomyces*, *Campylobacter*, *Fusobacterium*, *Leptotrichia*, *Porphyromonas*, *Prevotella*, *Streptococcus* and *Tannerella* (**Figure 2D**).

In periodontitis group, we explored the differential taxa among stages of periodontitis. In both types of oral samples, no significant difference was found in the alpha diversity analysis and beta diversity analysis among the different stages of periodontitis. There were no difference in the taxa among stages of periodontitis (**Figure S4B, S4C**, *P* > 0.05).

### 3.3 Microbial alterations in different diseases

**Figure 3:**
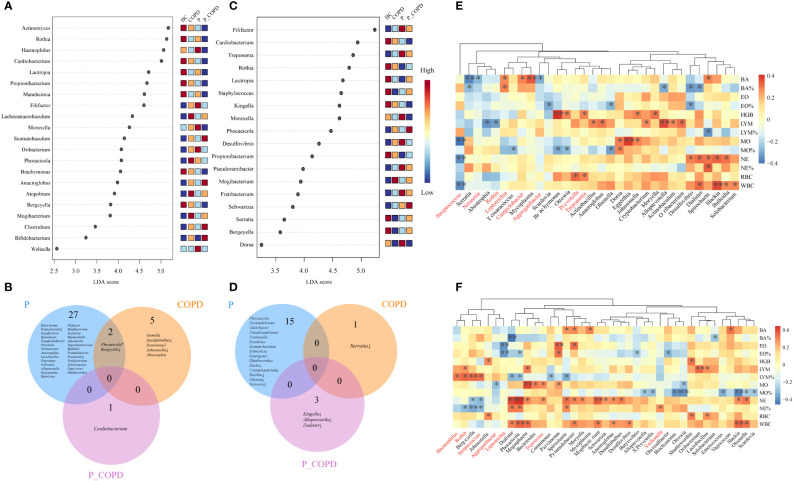
Linear discriminant analysis of effect size (LEfSe) of oral microbiota at the genus level enriched in healthy controls (HC), periodontitis (P) group, chronic obstructive pulmonary disease (COPD) group and comorbid diseases (P_COPD) group. LDA score (log10) <2, *P* < 0.05) **(A)** In subgingival plaque samples. **(C)** In gingival crevicular fluid samples. DESeq2 analysis of oral microbiota. Oral microbiota with significantly difference from the diseased groups compared with healthy controls **(B)** in subgingival plaque samples, **(D)** in gingival crevicular fluid samples. * The relative abundances of *Phocaeicola* was more abundant in CP group, while the relative abundance of *Phocaeicola* in COPD group was lower than HC groups. ↓, decreased. Spearman’s coefficient calculated between oral microbiota and clinical indicators. The taxa analyzed were the top 20 genera in terms of abundance **(E)** in subgingival plaque samples, **(F)** in gingival crevicular fluid samples. Black stars within heatmap boxes indicate significant results (*: *P* ≤ 0.05, **: *P* ≤0 .01), Benjamini–Hochberg adjustment for multiple comparisons.

To further identify the differential taxa among these groups, LEfSe and DESeq2 analyses were conducted. According to the LEfSe analysis, in the SP samples, eight genera were predominant in the HC group, including *Actinomyces*, *Bergeyella*, *Brachymonas*, *Cardiobacterium*, *Lautropia*, *Mannheimia*, *Propionibacterium* and *Rothia*. In contrast, the abundance of *Haemophilus*, *Filifactor*, and *Moraxella* increased in the periodontitis group. The abundance of *Atopobium* and *Lachnoanaerobaculum* were higher in the COPD group and the abundance of *Stomatobaculum*, *Anaeroglobus*, *Bifidobacterium*, and *Clostridium* were higher in the P_COPD group. (**Figure 3A**, LDA score (log10) >2, *P* < 0.05). According to DESeq2 analysis (**Table S1**), there were significant differences in the oral microbiota of the three diseased groups in the SP samples but no common change among these groups. Twenty-nine genera were predominant in the periodontitis group, including *Filifactor*, *Mogibacterium*, *Scardovia*, *Murdochiella* and *Odoribacter. Abiotrophia* and *Gemella* were more abundant in the COPD group and the abundance of *Cardiobacterium* was higher in the P_COPD group. The abundance of *Bergeyella* decreased in the periodontitis and COPD groups. The abundance of *Pasteurella* and *Propionicicella* decreased in the periodontitis group. The abundance of *Desulfobulbus*, *Soonwooa* and *Johnsonella* decreased in the COPD group (**Figure 3C**).

According to LEfSe analysis, in the GCF samples, the abundance of eight genera: *Bergeyella*, *Cardiobacterium*, *Kingella*, *Lautropia*. *Propionibacterium*, *Rothia*, *Serratia* and *Staphylococcus* were more abundant in the HC group. *Desulfovibrio*, *Dorea*, *Filifactor*, *Fretibacterium*, *Moraxella*, *Pseudoramibacter* and *Treponema* were more abundant in the P group, while the abundance of *Mogibacterium* increased in the COPD group. The abundance of *Phocaeicola* and *Schwartzia* was higher in the P_COPD group. (**Figure 3B**, LDA score (log10) >2, *P* < 0.05). According to DESeq2 analysis (**Table S2**), there were significant differences in the oral microbiota of the three diseased groups, but no common changes were observed among these groups. Fifteen genera were predominant in the periodontitis group: *Pasteurella*, *Phocaeicola*, *Pseudoramibacter*, *Pseudoramibacte*r, *Pyramidobacter*, *Scardovia*, *Schwartzia*, *Shuttleworthia*, *Slackia*, *Stomatobaculum* and *Synergistes*. The abundance of *Corynebacterium*, *Bacillus*, *Ottowia* and *Neisseria* decreased in the periodontitis group. The abundance of *Serratia* decreased in the COPD group. The abundance of *Kingella*, *Alloprevotella* and *Dialister* decreased in the P_COPD group (**Figure 3D**).

### 3.4 Association between genera and blood routine indicators

The observed links between the respiratory microbial community and disease prompted us to examine the interactions between the taxa and their clinical features. The taxa analyzed were the top 20 genera regarding abundance. For SP samples, the relative abundance of *Streptococcus* was negatively correlated with neutrophil, white blood cell and monocyte counts. The relative abundance of *Rothia* was negatively correlated with lymphocyte counts. The relative abundance of L*eptotrichia* and *Campylobacter* were positively correlated with the basophil counts, and the relative abundance of *Aggregatibacter* and *Neisseria* were negatively correlated with the basophil counts (**Figure 3E**). In the GCF samples, the relative abundance of *Rothia*, *Streptococcus*, and *Haemophilus* was positively correlated with the lymphocyte percentages. The relative abundance of *Streptococcus* and *Rothia* was negatively correlated with the neutrophil percentages, and the relative abundance of *Veillonella* was positively correlated with neutrophil percentages. The relative abundance of *Treponema* was positively correlated with neutrophil counts. In addition, the relative abundance of *Leptotrichia* was negatively correlated with the eosinophil percentages (**Figure 3F**).

### 3.5 Potential function of oral microbiome

We analyzed the predicted phenotypes based on taxonomic classification using BugBase. In different sample types, the relative abundance of aerobic bacteria in the HC group was higher than that in the diseased groups (**Figure S2A**, **S2F**). In comparison, the relative abundance of anaerobic bacteria was lower in the HC group than that in the diseased groups (**Figure S2B**, **S2G**). The ability to form biofilms in the HC group was greater than that in the diseased groups (**Figure S2C**, **S2H**). The potential pathogenicity in the HC group was lower than that in the diseased groups in the SP samples (**Figure S2D**). In the GCF samples, the potential pathogenicity in the P group was lower than that in the other groups (**Figure S2I**). In addition, in the SP samples, the relative abundance of gram-positive bacteria in the HC group was higher than that in the other diseased groups, whereas gram-negative bacteria showed the opposite trend (**Figures S2E**, **S2J**).

**Figure 4:**
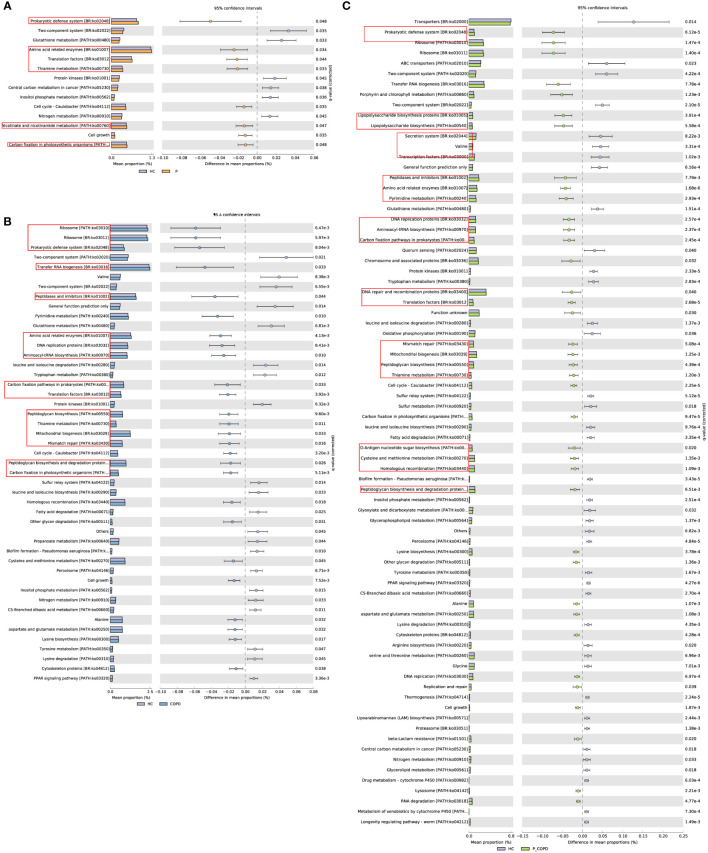
PICRUSt analysis in the KEGG pathways. Functional predictions for the oral microbiome of the diseased groups and healthy control group. Significant KEGG pathways at level 3 for the oral microbiome of the diseased groups and healthy control group in subgingival plaque samples were identified by STAMP software. Bar chart showing the functional difference (corrected *p*-value < 0.05) between periodontitis **(A)**, chronic obstructive pulmonary disease **(B)** and comorbid diseases **(C)** versus healthy controls. PICRUSt, Phylogenetic Investigation of Communities by Reconstruction of Unobserved States; KEGG, Kyoto Encyclopedia of Genes and Genomes.

Through PICRUSt2, putative biological functions of the microbiota of the four groups were illustrated. No significant differences were observed between the GCF samples. As shown in **Figure 4A**, the periodontitis group exhibited significantly enriched metabolism of cofactors and vitamins (thiamine metabolism, nicotinate and nicotinamide metabolism), translation, protein families: genetic information processing (translation factors), amino acid related enzymes, and carbon fixation in photosynthetic organisms. The COPD group showed significantly enriched protein families: genetic information processing (transfer RNA biogenesis, ribosome, mitochondrial biogenesis, DNA replication proteins, translation factors), translation (ribosome, aminoacyl-tRNA biosynthesis, RNA transport), replication and repair (homologous recombination, mismatch repair, DNA replication), protein families: metabolism (peptidases and inhibitors, amino acid related enzymes, peptidoglycan biosynthesis and degradation proteins), glycan biosynthesis and metabolism(peptidoglycan biosynthesis, other glycan degradation, other types of O-glycan biosynthesis and mannose type O-glycan biosynthesis) (**Figure 4B**). The P_COPD group showed significantly enriched protein families: genetic information processing (DNA repair and recombination proteins, transfer RNA biogenesis, ribosome, chromosome and associated proteins), protein families: metabolism (amino acid related enzymes, peptidases and inhibitors, peptidoglycan biosynthesis and degradation proteins), translation (ribosome, aminoacyl-tRNA biosynthesis, RNA transport), glycan biosynthesis and metabolism(peptidoglycan biosynthesis, other glycan degradation, lipopolysaccharide biosynthesis), metabolism of cofactors and vitamins(lipoic acid metabolism, porphyrin and chlorophyll metabolism, riboflavin metabolism, thiamine metabolism) and other functions (**Figure 4C**).

The HC group showed significantly enriched signal transduction (two-component system), lipid metabolism (biosynthesis of unsaturated fatty acids), and metabolism of other amino acids (glutathione metabolism, phosphonate and phosphinate metabolism), compared to the other three groups (**Figure S3**).

## 4 Discussion

The alteration of oral microecosystem in patients with systemic diseases has been the subject of intense research for several years (Thomas et al., 2021). An increasing amount of evidence from microbiological studies indicates a significant ecological connection between oral microecosystems, periodontitis and COPD (Wu et al., 2022). Here we explored the oral microbiota of SP and GCF in periodontitis, COPD, comorbid patients, and healthy controls. In this study, we collected oral microbial samples of two types. Compared with the GCF, differences in the microbial community compositions of SP more clearly expressed the varieties of oral microecology in periodontitis and COPD, indicating that it may be more appropriate for reflecting the difference of subgingival microbiota between periodontitis and COPD.

This study observed higher bacterial richness in individuals suffering from periodontitis in the two types of oral samples, suggesting that microbial dysbiosis were existed in the process of periodontitis (Lin et al., 2020).

Using LEfSe analysis, we identified differentially abundant genera associated with different diseases. In the present study,periodontitis group had a higher abundance of *Desulfovibrio*, *Filifactor*, *Fretibacterium*, *Moraxella*, *Odoribacter*, *Pseudoramibacter*, *Pyramidobacter*, *Scardovia*, *Shuttleworthia* and *Treponema* in the two types of samples. *Pseudoramibacter, Pyramidobacter, Scardovia, Shuttleworthia* and *Desulfovibrio* have been recognized as periodontitis-associated genera (Colombo et al., 2009; Huynh et al., 2017; Shi et al., 2018). *Treponema denticola*, *Porphyromonas gingivalis*, and *Tannerella forsythia* have been designated as ‘red-complex’ periopathogens and have shown a strong association with periodontitis (Darveau, 2010). . It has been reported that patients with COPD tend to have relatively higher ranked means of *Treponema denticola* than healthy participants (Zhou et al., 2020). The COPD group had a higher abundance of *Mogibacterium* in both sample types. The abundance of *Abiotrophia, Atopobium, Gemella* and *Phocaeicola* also increased in SP samples. In the previous studies, *Abiotrophia*, *Atopobium*, *Mogibacterium* and *Phocaeicola* were common periodontitis-associated genera (Mikkelsen et al., 2000; Camelo-Castillo et al., 2015; Zhang et al., 2015; Coretti et al., 2017). Besides, we found that no study has adequately described the connection and characteristics of these genera in patients with COPD; *Mogibacterium* is associated with persistent generalized disease (Nibali et al., 2020). . Patients in the P_COPD group had high proportions of the genera *Anaeroglobus, Bifidobacterium* and *Clostridium* in SP samples and *Phocaeicola* and *Schwartzia* in the GCF samples. *Phocaeicola* and *Schwartzia* have been previously identified in periodontitis (Camelo-Castillo et al., 2015). *Bergeyella*, *Lautropia*, *Rothia*, *Propionibacterium* and *Cardiobacterium* were more abundant in the healthy participants. *Bergeyella* was considered as putative periodontal protectors in periodontal swabs from the participants (Zorina et al., 2014). *Lautropia mirabilis*, *Propionibacterium propionicum*, *Rothia dentocariosa/mucilagenosa* and *Cardiobacterium hominis* were significantly more prevalent in the healthy group than in the periodontitis patients (Colombo et al., 2009; Ikeda et al., 2020).

This study and observed the association between genera and blood routine indicators. The inflammatory mediators produced by pathogenic microorganisms promote the development of periodontal inflammation and enter the systemic blood circulation, which affects the inflammatory development of systemic diseases (Kumar, 2017). Here we explored the association between genera and blood routine indicators. *Anaeroglobus geminatus* is positively correlated with different lipid mediators which are related to the inflammatory process of periodontitis (Lee et al., 2021). We also observed that the relative abundance of *Anaeroglobus* was positively correlated with lymphocyte counts, indicating that dysbiosis of periodontal-associated microorganisms may accelerate the process of inflammatory between periodontitis and COPD. In our study, the relative abundance of *Treponema* and *Filifactor* were significantly increased in periodontitis group. The relative abundance of *Treponema* was positively correlated with neutrophil counts in GCF samples. In the previous study, *Filifactor, Treponema, and Fretibacterium*, which were more abundant in patients with periodontitis, were proved connected with inflammatory mediators (Lundmark et al., 2019). *Treponema* sp. and cytokines chitinase 3-like 1, sIL-6Rα, sTNF-R1, and gp130/sIL-6Rβ were positively correlated, a negative correlation was identified between IL-10 and *Filifactor alocis*. We discovered that the relative abundance of *Streptococcus* and *Rothia* was negatively correlated with the neutrophil percentages in the GCF samples. In previous study, as a common microorganism of the oral cavity, the presence of *Rothia mucilaginosa* in the lower airways potentially mitigates inflammation (Rigauts et al., 2022). The levels of *Rothia* and *Streptococcus* were significantly lower in oropharyngeal microbiota composition, in both the COVID-19 and flu patients than in the healthy control group, which indicated oropharyngeal microbiota composition may influence the severity of the disease and the progression of inflammation (Ma et al., 2021).The results of our study were similar to previous studies, which partly proved that alterations of periodontal-associated microorganisms may impact the progression of inflammation in respiratory disorders, and indicated that the specific high-abundance bacteria in the four groups may have vital clinical significance for the early diagnosis and treatment of periodontitis and COPD.

The differences in metabolic pathways and functions caused by alteration of microbiota were evident in the SP samples. We performed functional predictions based on the KEGG database. Genetic information processing and translation were significantly different between the periodontitis, COPD, and P_COPD groups. It is worth noting that the functions related to bacteria proliferation were higher in these groups. This may partly explain the higher diversity and density of patients with periodontitis and COPD (Shi et al., 2021). The metabolism of cofactors and vitamins was significantly enriched in the periodontitis and P_COPD groups. Nicotinate and nicotinamide metabolism is associated with the important metabolic pathways in the keystone periodontal pathogen, *Porphyromonas gingivalis *(Hutcherson et al., 2016). Thiamine is essential for several important enzymes involved in carbohydrate metabolism and associated with the key nutrient for *Treponema denticola* survival (Bian et al., 2015). The metabolism of glutathione, phosphonate and phosphinate was significantly decreased in the periodontitis, COPD, and P_COPD groups similar to observations from previous studies. Glutathione is an antioxidant that can moderate host cell damage and reduce inflammatory response (Ghezzi, 2011). *Treponema denticola* is connected to the catabolism of glutathione to H2S (Chu et al., 2020) and the diseased periodontal pockets of periodontitis patients have lower glutathione levels than healthy sites. Glutathione metabolism may be a key pathway for inflammatory damage in COPD.

This study had several limitations. First, compared to the healthy and periodontitis groups, fewer female individuals were recruited for the COPD, and P_COPD groups because of the difficulty in recruiting older female individuals with COPD. Second, this study was not a longitudinal study which limited the exploration of variations in the oral microbiota during disease progression. Then, the detectable microbial diversity is limited in our sample types, we used 16S rRNA gene amplification which limited our ability to identify specific bacteria at the species level. We will refine this in subsequent studies. Finally, the lower airway microbiota samples were not collected in this study. Studies on association between periodontal bacteria and bacteria in the lower airway are insufficient.

## 5 Conclusion

The present study discovered that the presence of periodontitis and COPD altered the compositions and functional characterization of oral microbiomes. These diversities in microecology were correlated with the pathological change in diseases. These results may have vital clinical significance in the screening and treatment of individuals with periodontitis and COPD.

## Data availability statement

The datasets presented in this study can be found in online repositories. The names of the repository/repositories and accession number(s) can be found below: NCBI, BioProject ID: PRJNA910319.

## Ethics statement

The present study was approved by the ethics committee of Linfen Central Hospital (Ethics Approval No. 2021-42-1) and was performed in accordance with the Declaration of Helsinki. Written informed consent was obtained from all participants prior to clinical data collection and sampling.

## Author contributions

SL, YHo and ZG designed the research project. GX, MC, YZ, QC and YQ practiced sample collection. SL, XM, JX and MJ performed DNA extraction and sequencing data analysis. SG, YHo and ZG conducted experiments and contributed significantly to analysis and manuscript preparation. SL, YHe and WY performed the data analyses and wrote the manuscript. XC, WM and NL helped perform the analysis with constructive discussions. All authors contributed to manuscript revision, read, and approved the submitted version.
