# Supplementary material for: Oral microbial dysbiosis in patients with periodontitis and chronic obstructive pulmonary disease
Source: Front Cell Infect Microbiol. 2023 Feb 9;13:1121399. doi: 10.3389/fcimb.2023.1121399 (PMC9948037; doi:10.3389/fcimb.2023.1121399)
Supplement: Supplementary file 1 [file DataSheet_1.docx]

Supplementary Material

Oral microbial dysbiosis in patients with periodontitis and chronic obstructive pulmonary disease

Authors: Siqin Liu^1#^, Guofang Xie^2#^ Meifeng Chen^3#^, Yukun He^4^, Wenyi Yu^4^, Xiaobo Chen^2^, Weigang Mao^2^, Nanxia Liu^2^, Yuanjie Zhang^2^, Qin Chang^3^, Yingying Qiao^3^, Xinqian Ma^4^, Jianbo Xue^4^, Mengtong Jin^5^, Shuming Guo^6*^, Yudong Hou^1*^, Zhancheng Gao^4*^

Affiliation:

1. School of Stomatology, Binzhou Medical University, Yantai, China.

2. Department of Stomatology, Linfen Central Hospital, Linfen, China.

3. Department of Respiratory and Critical Care Medicine, Linfen Central Hospital, Linfen, China.

4. Department of Respiratory and Critical Care Medicine, Peking University People's hospital, Beijing, China.

5.Department of Science and Education, Linfen Central Hospital, Linfen, China.

6.Nursing department, Linfen Central Hospital, Linfen, China

#These authors share first authorship.

*Correspondence to:

Shuming Guo

Nursing department, Linfen Central Hospital, Linfen, China.

041000

Tel 86-0357-2399000

Fax 86-0357-2399002

Email kyzx@linfench.com

Yudong Hou

School of Stomatology, Binzhou Medical University, Yantai, China

264000

Tel 86-0535-6913987

Fax 86-0535-6913987

Email bycgk@126.com

Zhancheng Gao

Department of Pulmonary and Critical Care Medicine, Peking University People’s hospital, Beijing, China

100044

Tel 86-10-88324866

Fax 86-10-68318386

Email zcgao@bjmu.edu.cn

**Supplementary Table 1. Permutational multivariate analysis of variance (Adonis test) (999 permutations) were conducted using the adonis2 function in the “vegan” R package to analyse the significance of the community differences.**

| pairs | R^2^ | *p*.value | *p*.adjusted |
| --- | --- | --- | --- |
| In subgingival plaque samples. | | | |
| HC vs P | 0.06742238 | 0.003 | 0.009 |
| HC vs COPD | 0.05005678 | 0.005 | 0.01 |
| HC vs P_COPD | 0.07565631 | 0.001 | 0.006 |
| P vs COPD | 0.0253931 | 0.208 | 0.2496 |
| CP vs P_COPD | 0.0280725 | 0.13 | 0.195 |
| COPD vs P_COPD | 0.01473564 | 0.575 | 0.575 |
| In gingival crevicular fluid samples | | | |
| HC vs P | 0.06039775 | 0.005 | 0.014 |
| HC vs COPD | 0.05252385 | 0.007 | 0.014 |
| HC vs P_COPD | 0.09205031 | 0.001 | 0.006 |
| P vs COPD | 0.02814596 | 0.154 | 0.1848 |
| P vs P_COPD | 0.03829939 | 0.019 | 0.0285 |
| COPD vs P_COPD | 0.0139369 | 0.674 | 0.674 |

*p*.adjusted is correction of *P* value.

**Supplementary Table 2. Significant microbiota in subgingival plaque samples compared with diseased groups and healthy controls**

|  | Log2FC | *p*-adj |
| --- | --- | --- |
| periodontitis |  |  |
| *Filifactor* | 11.27269 | 1.63E-09 |
| *Mogibacterium* | 11.9418 | 4.78E-06 |
| *Scardovia* | 29.99926 | 4.79E-06 |
| *Murdochiella* | 29.07887 | 8.23E-06 |
| *Odoribacter* | 27.53271 | 2.50E-05 |
| *Phocaeicola* | 11.75011 | 2.50E-05 |
| *Peptostreptococcus* | 7.227821 | 0.000109 |
| *Bulleidia* | 24.36923 | 0.000227 |
| *Pyramidobacter* | 24.21815 | 0.000227 |
| *Pasteurella* | -23.2524 | 0.000427 |
| *Bergeyella* | -4.42024 | 0.00095 |
| *Fretibacterium* | 5.985712 | 0.001203 |
| *Solobacterium* | 21.24712 | 0.001401 |
| *Vagococcus* | 20.61549 | 0.002092 |
| *Shuttleworthia* | 20.45382 | 0.002183 |
| *Eubacterium* | 3.980127 | 0.007251 |
| *Propionicicella* | -18.42 | 0.00729 |
| *Desulfovibrio* | 17.91213 | 0.008299 |
| *Spirochaeta* | 17.876 | 0.008838 |
| *Pseudoramibacter* | 17.54351 | 0.009395 |
| *Prevotella* | 1.810862 | 0.009546 |
| *Oribacterium* | 8.333237 | 0.010422 |
| *Anaeroglobus* | 14.07844 | 0.018374 |
| *Lactobacillus* | 16.18564 | 0.018374 |
| *Treponema* | 2.878011 | 0.018466 |
| *Veillonella* | 2.193754 | 0.025657 |
| *Alloprevotella* | 4.439662 | 0.03485 |
| *Mycoplasma* | 7.261287 | 0.03813 |
| *Rubrivivax* | 14.40466 | 0.03813 |
| chronic obstructive pulmonary disease | |  |
| *Bergeyella* | -9.04466 | 0.004606 |
| *Phocaeicola* | -13.5216 | 0.012756 |
| *Gemella* | 5.36291 | 0.018665 |
| *Desulfobulbus* | -10.3276 | 0.026169 |
| *Soonwooa* | -28.9847 | 0.034795 |
| *Johnsonella* | -8.13364 | 0.045282 |
| *Abiotrophia* | 14.36545 | 0.045282 |
| comorbid diseases |  |  |
| *Cardiobacterium* | 14.43349 | 0.000383 |

*Log2FC (log2 fold change) represents the ratio of two groups based log2. *p*-adj is correction of *P* value.

**Supplementary Table 3. Significant microbiota in gingival crevicular fluid samples compared with diseased groups and healthy controls**

|  | Log2FC | *p*-adj |
| --- | --- | --- |
| periodontitis |  |  |
| *Phocaeicola* | 18.17787387 | 3.67E-10 |
| *Bacillus* | -30 | 8.12E-06 |
| *Pyramidobacter* | 27.43435721 | 4.93E-05 |
| *Odoribacter* | 24.35442924 | 0.000546667 |
| *Pseudoramibacter* | 23.20461545 | 0.000712434 |
| *Pasteurella* | 23.3674166 | 0.000712434 |
| *Scardovia* | 23.51568378 | 0.000712434 |
| *Stomatobaculum* | 12.71577697 | 0.00079628 |
| *Schwartzia* | 13.09651395 | 0.00277816 |
| *Ottowia* | -6.008837867 | 0.002880799 |
| *Synergistes* | 20.49671588 | 0.003275652 |
| *Shuttleworthia* | 19.5113025 | 0.005737697 |
| *Neisseria* | -1.977003126 | 0.006388967 |
| *Slackia* | 18.42713272 | 0.009723567 |
| *Corynebacterium* | -1.792888837 | 0.045902814 |
| chronic obstructive pulmonary disease | |  |
| *Serratia* | -20.95922072 | 4.42E-10 |
| comorbid diseases |  |  |
| *Kingella* | -21.46239179 | 2.88E-08 |
| *Alloprevotella* | -14.88109724 | 7.75E-05 |
| *Dialister* | -14.46371702 | 0.00278854 |

*Log2FC (log2 fold change) represents the ratio of two groups based log2. *p*-adj is correction of *P* value.


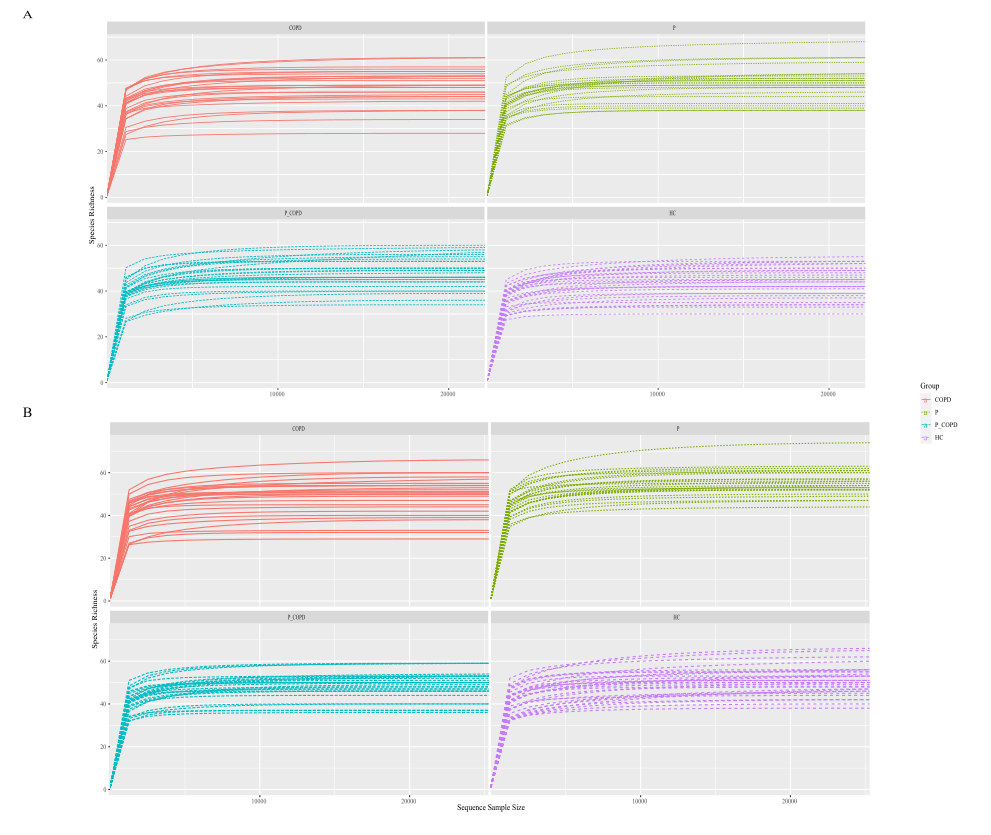


**Supplementary Figure 1. Rarefaction curves of all samples.** Rarefaction curve of estimated OTUs in the 16S rRNA libraries. (A)In subgingival plaque samples. (B) In gingival crevicular fluid samples.


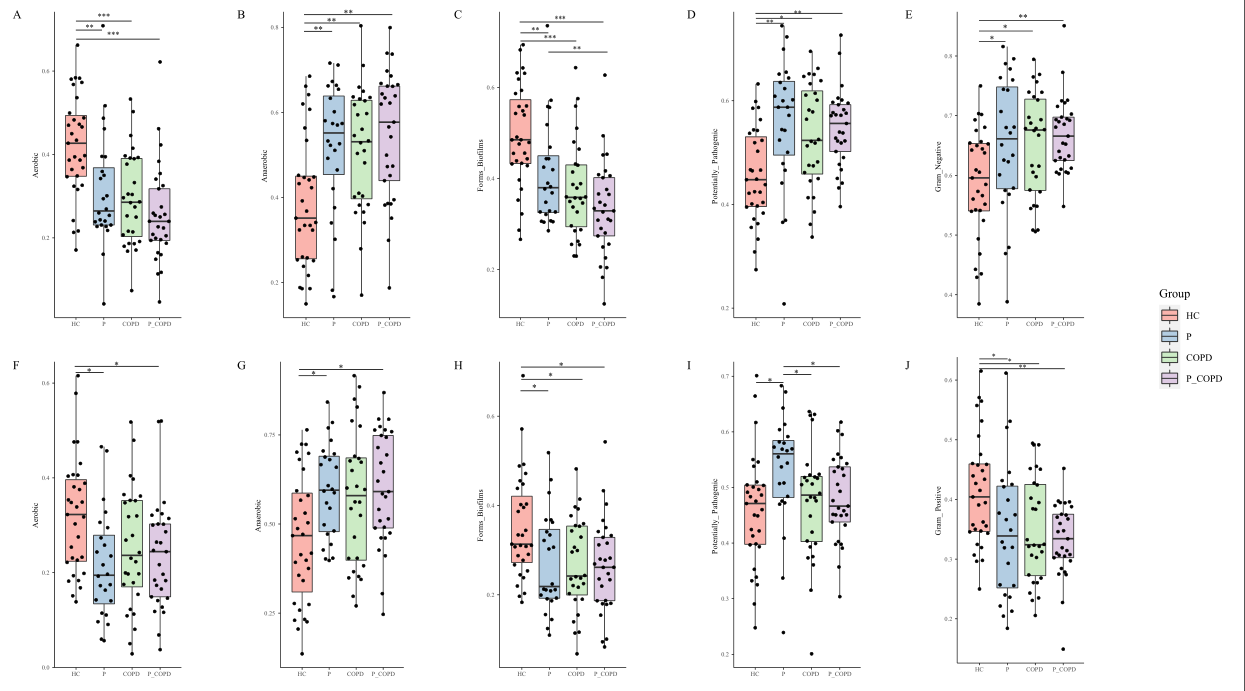


**Supplementary Figure 2. BugBase predicted microbial community phenotypes and the corresponding bacterial contributions.** In subgingival plaque samples (A) Relative abundance of aerobic bacteria. (B) Relative abundance of anaerobic bacteria. (C) Biofilm formation. (D) Pathogenic risk. (E) Relative abundance of gram negative bacteria. (J) Relative abundance of gram positive bacteria. In gingival crevicular fluid samples (F) Relative abundance of aerobic bacteria. (G) Relative abundance of anaerobic bacteria. (H) Biofilm formation. (I) Pathogenic risk.

**P* < 0.05, ***P* < 0.01, ****P* < 0.001


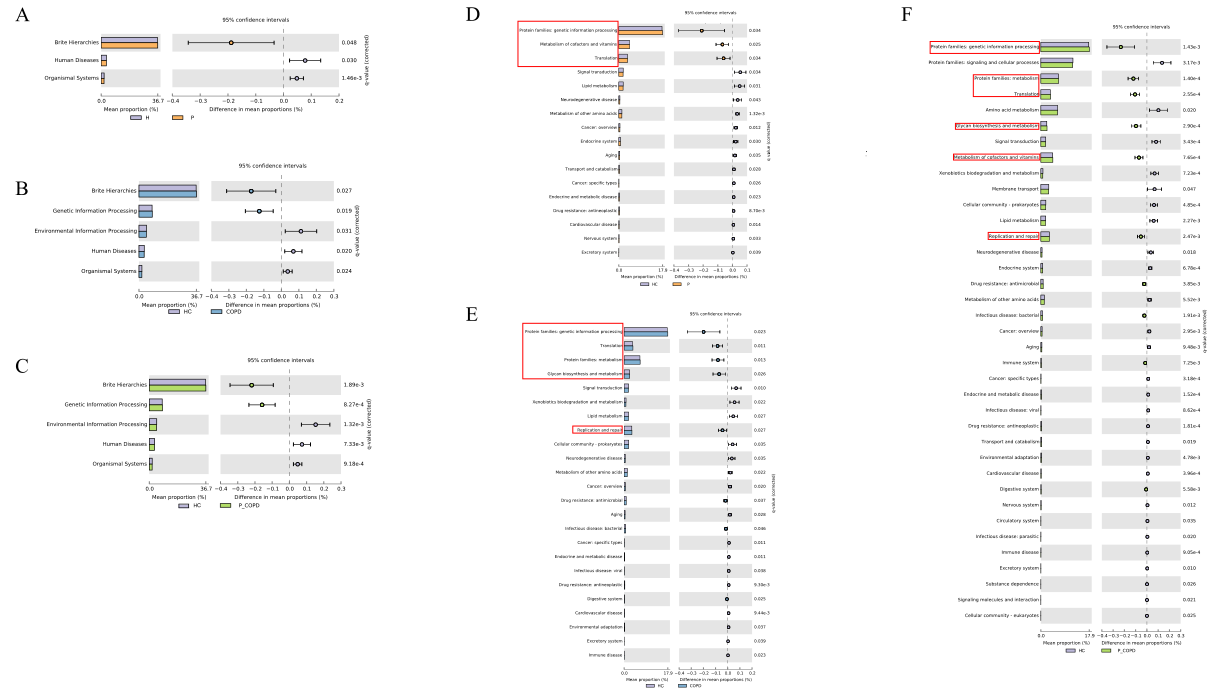


**Supplementary Figure 3. PICRUSt analysis in the KEGG pathways.** Functional predictions for the oral microbiome of the diseased groups and healthy control group. Significant KEGG pathways at level 1 and level 2 for the oral microbiome of the diseased groups and healthy control group in subgingival plaque samples were identified by STAMP software. Bar chart showing the functional difference (corrected *p*-value < 0.05) between periodontitis (A,D), chronic obstructive pulmonary disease (B,E) and comorbid diseases (C,F) versus healthy controls.

PICRUSt, Phylogenetic Investigation of Communities by Reconstruction of Unobserved States; KEGG, Kyoto Encyclopedia of Genes and Genomes.
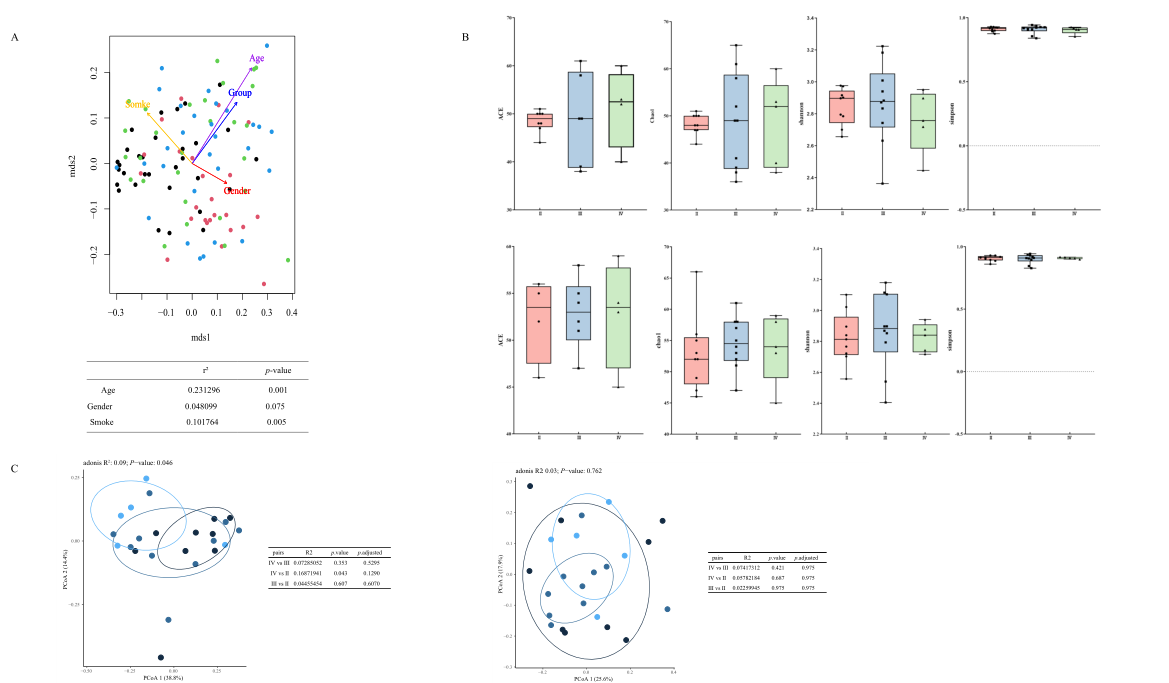


**Supplementary Figure 4. Correction of confounding factors.** (A)Envfit functions were used to explore the host factors associated with the microbial community and determine the confounding factors**. Alpha diversity analysis among stages of periodontitis(Ⅱ,Ⅲ,Ⅳ).** (B)Chao1, Shannon, abundance-based coverage estimator (ACE) and Simpson indices of each stage, in subgingival plaque samples and gingival crevicular fluid samples. No significant difference was found in the alpha diversity analysis among the different stages of periodontitis. **Principal coordinate analysis (PCoA) among stages of periodontitis(Ⅱ,Ⅲ,Ⅳ).** (C) No significant difference was found in the beta diversity analysis among the different stages of periodontitis.
